# The Quality of Life and Satisfaction with Continuous Glucose Monitoring Therapy in Children under 7 Years of Age with T1D Using the rtCGM System Integrated with Insulin Pump—A Caregivers Point of View

**DOI:** 10.3390/s21113683

**Published:** 2021-05-25

**Authors:** Ewa Rusak, Natalia Ogarek, Karolina Wolicka, Anna Mrówka, Sebastian Seget, Magdalena Kuźnik, Przemysława Jarosz-Chobot

**Affiliations:** 1 Department of Children’s Diabetology, Medical University of Silesia, 40-752 Katowice, Poland; sebastian.seget@sum.edu.pl (S.S.); przemka1@o2.pl (P.J.-C.); 2 Students’ Scientific Association at the Department of Children’s Diabetology, Medical University of Silesia, 40-752 Katowice, Poland; n.ogarek2@gmail.com (N.O.); kwolicka95@gmail.com (K.W.); a.mrowka@onet.eu (A.M.); 3 The Upper Silesian Child Health Centre, 40-752 Katowice, Poland; kuznik.magdalena@o2.pl

**Keywords:** diabetes type 1 children, quality of life, continuous glucose monitoring

## Abstract

Quality of life (QoL) is an important parameter that affects the choice of therapy. Assessment of QoL and satisfaction with therapy using the rtCGM in children with T1D aged < 7 years was conducted. The study group consisted of 38 children with T1D aged < 7 years (34% aged 2–4, 66% aged 5–7 years), HbA1c: 6.53 ± 0.63%, duration of diabetes: 2.6 ± 1.6 years, treated with an rtCGM-augmented insulin pump for 1.92 ± 1.15 years. Two anonymous surveys were conducted: a. PedsQL3.0 diabetes standardized questionnaire—QoL assessment among age groups: 2–4/5–7 years. b. An original survey assessing the CGM use satisfaction. The mean scores in PedsQL3.0: communication 75%, worries 30%, treatment 70%, and problems associated with diabetes 65%. The QoL scale is: 0–19% very low, 20–39% low, 40–59% moderate, 60–79% high, 80–100% very high. The most frequently reported concerns were long-term diabetes complications and prick pain. Satisfaction with CGM use was high (68% in group aged 5–7 and 92% 2–4 years). Twenty-seven (71%) caregivers confirmed the positive effect of CGM on sleep. During the use of rtCGM a high quality of life was reported, and the quality of sleep in their caregivers was increased.

## 1. Introduction

Type 1 diabetes mellitus (T1D) is a chronic disease that causes an extraordinary burden on children and their caregivers [1].

Both the disease and the restrictions associated with treatment constitute a limitation on the daily life of children and their families, undoubtedly affecting its quality [2,3,4,5,6].

The dynamic progress of technology in the field of diabetology makes it possible to improve the quality of life and minimize the disparity between affected children and healthy ones [7]. The use of continuous glucose monitoring systems (CGM) to measure glucose concentrations and illustrate its trends has proven to be a breakthrough in everyday clinical practice. CGM allows caregivers to conduct real-time therapy for diabetes with parameters of glycemic variability, which is important in the prevention of acute and chronic complications of diabetes, such as nephropathy, retinopathy, neuropathy, stroke, and myocardial infarction [2,8,9].

CGM systems may interact with insulin pumps (insulin pumps integrated with a sensor with the function of low-glucose suspension or in low-glucose prediction and resumption of insulin delivery)—this enables automation of insulin dosing, or the system and pump may be used separately [10,11,12].

Studies confirm that the use of CGM systems reduces the time above and below glucose target range compared to self-monitoring of blood glucose (SMBG), reduces the variability of blood glucose, improves the glycated hemoglobin (HbA1c), which reflects average blood glucose concentration during the last 3 months and reduces the number of hypoglycemic episodes [1,2,13].

These benefits improve the comfort of life: they reduce the stress associated with changes in glucose concentration and episodes of hypoglycemia, enabling more effective control of the disease and normal life activity without stigmatizing the disease [14,15,16,17]. 

Nevertheless, the lack of CGM precision, discomfort of wearing, and malfunctions are known disadvantages connected with CGM use, which may affect the quality of life.

The youngest children are completely dependent on caregivers for disease control [6]. In order to draw attention to the unique role and clear challenges of using a CGM system, the authors have undertaken this study.

The study was conducted to assess the quality of life and satisfaction in the youngest children suffering from T1D using the personal insulin pump integrated with the real-time CGM (rtCGM) system from a caregiver’s point of view.

## 2. Materials and Methods

The study group consisted of 38 children under 7 years of age with well-controlled T1D (mean HbA1c: 6.53 ± 0.63%), monitoring glycemia through an rtCGM system integrated with an insulin pump. Children as a heterogeneous group in terms of psychomotor development and the degree of independence were divided into two age groups: 2–4 and 5–7 years old, 34% and 66%, respectively. The study participants were recruited in the Outpatient Clinic of the Department of Children’s Diabetology, Medical University of Silesia, Katowice, Poland. If a child met the enrolling criteria, participation in the study was offered to caregivers. The questionnaires were filled out voluntarily only by interested individuals.

The study was conducted between February 2019 and February 2020.

Clinical data are presented in Table 1. All study participants were treated with an insulin pump (MiniMed 640G or MiniMed Paradigm VEO-754, Medtronic, Fridley, MN, USA) integrated with the rtCGM system. Thirty-one point five percent were treated with the rtCGM system from the moment of the disease diagnosis; the remaining children, before the application of rtCGM, were self-controlled by measuring the glycemia from the fingerstick. Sensors were inserted only into the area of the back of the upper arm in all children.

Caregivers of children responded to 2 anonymous questionnaires: standardized PedsQL 3.0 Diabetes questionnaire and the author’s questionnaire. The standardized PedsQL 3.0 Diabetes questionnaire is available in 3 versions, depending on the age groups: 0–2 years, 2–4 years, 5–7 years. It was used to assess the quality of life in children with diabetes, downloaded from the Mapi Research Trust website (https://eprovide.mapi-trust.org, http://www.pedsql.org) accessed on 16 February 2019 [18].

The author’s questionnaire consisted of 14 questions—3 open, 3 closed, and 8 semi-open. The author’s questionnaire assessed the impact of rtCGM on changes in daily life and the satisfaction of continuous glycemic control (Supplementary Materials).

This study was approved by The Institutional Ethical Committee of The Medical University of Silesia, Katowice, Poland (KNW/0022/KB/18/19). 

## 3. Results

The average number of points in the Quality of Life Survey (PedsQL 3.0) in each category, the average number of points in the Quality of Life Survey (PedsQL 3.0), and the scale of quality of life are shown in Table 2, Table 3 and Table 4. The results of the group of children in whom the CGM system had been applied since the beginning of the disease did not differ from the overall average (Table 4). 

Concerns about late complications of diabetes and injection pain (injections/vein puncture—except for fingerstick glycemia measurements and insulin injections) were the most frequently reported. In addition, anxiety about hypoglycemia and fear of treatment failure were reported (Table 5). 

On the basis of the author’s survey, the influence of CGM on changes in the daily life of the child and caregivers were assessed, along with the satisfaction with the use of continuous glycemic control. 

**Figure 1:**
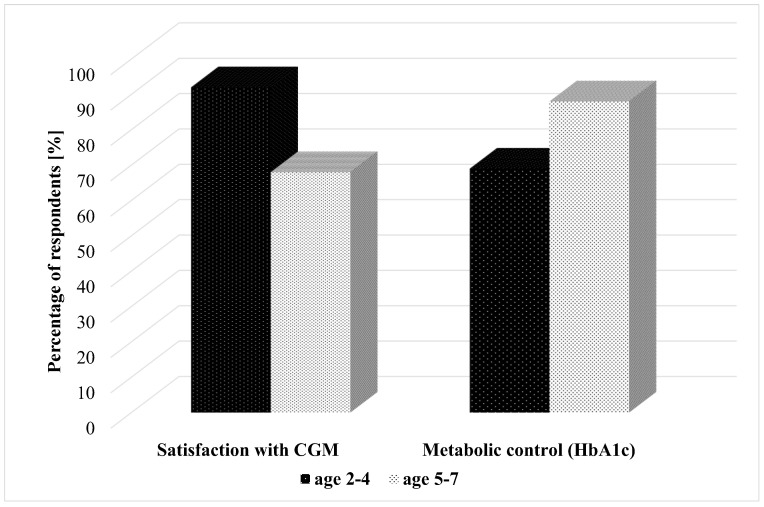
Satisfaction of caregivers with CGM usage and metabolic control.

Satisfaction with CGM was reported by 68% of caregivers of 5–7 year-olds and the majority—92% of 2–4 year-olds. The HbA1c < 7% in the youngest children—2–4-year-olds, was 69%, and in 5–7 years—88% (Figure 1). 

According to 65% of the surveyed, kindergarten staff were calmer when taking care of a child after using the CGM system. It was emphasized, however, that despite the application of CGM, the lack of proper childcare in the kindergarten sometimes forced parents to resign from professional work. This was one of the main reasons for disappointment with CGM. The dissatisfaction with CGM was also influenced by the incompatibility of readings between CGM and glucose meter measurements. 

Twenty-six percent of caregivers mentioned sleep alarms as a negative effect of the CGM system. However, 27 (71%) confirmed the positive effect of CGM on their sleep (“I sleep better, I am calmer”). Indeed, more often, the caregivers of children with good metabolic control answered this question positively (12% vs. 88%, *p* = 0.031).

During the use of the CGM system, in two cases out of 38 respondents, there was an episode of severe hypoglycemia. Slightly more than 50% of the respondents reported that a child was more engaged in glucose self-control. Only 18% reported that continuous alarms made the child no longer pay attention to them.

The use of the CGM system, in most cases, had no influence on undertaking sports activities. However, some caregivers reported the fear of damaging the equipment during sports. 

Seventy percent of the respondents reported a significant reduction in the fingerstick glycemia measurements (more than 50%) and a very high feeling of safety when using the CGM system in comparison to the period without CGM.

Apparently, there was also a positive influence on diet and spending free time (54% and 68% of respondents, respectively).

## 4. Discussion

Most of the published papers on insulin pump treatment with a CGM system focus on clinical parameters to evaluate metabolic control of diabetes. There are scarce and divergent reports on the psychological aspects and quality of life of the youngest group of patients. The particular nature of this age group results in difficulty in clearly expressing one’s needs and lack of independence in the personal pump operation. 

According to Hommel et al., treatment satisfaction is increased during CGM use in adults, while in school children, the use of CGM does not affect the quality of life. Furthermore, the metabolic control has improved significantly after the inclusion of the CGM system into the treatment, and the number of hypoglycemic episodes has decreased [5]. 

A review of the available literature showed that the overall quality of life of children with T1D is comparable with healthy children. Only a specific assessment of the quality of life associated with T1D revealed a number of concerns related to the complications and problems of everyday life with the disease [19]. In assessing quality of life, proper sleep remains a very important aspect, both in children and their caregivers. Night-time rest is disturbed by frequent alarms, the need to check blood glucose levels, and anxiety about hypoglycemia [20]. In a study conducted by Burckhardt et al., parameters assessed among caregivers of preschool children were also related to the parents’ sleep—better sleep was highlighted during CGM use [21]. In our analysis, we also noted an improvement in the sleep of caregivers. However, available information about the sleep of parents remains contradictory. A study conducted among caregivers of children aged 2–5 years showed an improvement in parents’ quality of life related to the improvement in children’s sleep associated with the use of CGM. The increase in the child’s sleeping time resulted from minimizing awakenings for nightly glycemia measurement. Interestingly, it appears that the night-time awakenings associated with the measurement of glycemia are more burdensome for parents than for children because the sleep quality of caregivers has not improved [22]. 

Hypoglycemia is one of the most frequent acute complications of insulin therapy which arouses anxiety. Severe hypoglycemia occurs in about 5–12% of children and is a serious problem [23,24]. It is the main indication for the use of CGM in the youngest children, which protects against an unexpected drop in blood glucose concentration. In our study, as many as 60% of caregivers reported anxiety about hypoglycemia. Daily management of diabetes, periprandial decisions, physical activity, insulin therapy itself—all these create the risk of hypoglycemia and its consequences. After severe hypoglycemia, a particular consequence is a psychological barrier which is an obstacle to achieving satisfactory metabolic control of diabetes [23].

Several studies have confirmed the reduction in hypoglycemia anxiety in children using CGM both separately and pump augmented [17,20,21]. Pre-hypoglycemic alarms are a warning. The caregiver can react before the occurrence of hypoglycemia or quickly during hypoglycemia. The new diabetology is based on reacting to glycemic trends, one step ahead, not to current glycemia. The alarm system warns against both hypoglycemia and hyperglycemia, sometimes generating many alarms during the day. As a consequence, the disadvantage is that children/caregivers adapt to the sound of an alarm that is too often triggered and do not respond to it. Therefore, it is extremely important to individualize the settings of the sensor so that one can really benefit from the possibilities that open up thanks to its use. In our survey, only 18% of caregivers reported that continuous alarms made the child no longer pay attention to them. 

The available data on the use of CGM revealed a high sense of freedom and confidence associated with the reduction in the fear of hypoglycemia, despite the anxiety reported at the same time—associated with the enormity of data that a parent receives. However, fewer worries, better control, and understanding of the disease were mainly emphasized [21]. These reports are consistent with the results of our original survey. The caregivers emphasized such advantages of the system as better glycemic control, facilitation of everyday functioning, increased sense of security, and the resulting increased possibilities for spending free time. However, attention was drawn to the fear of damaging the equipment during sports activities.

The previously described negative aspects of using CGM include, according to parents, the effects associated with wearing the device—problems with adhesion, skin irritation, or stigmatization of appearance [3]. In our study, apart from a single case (“peers’ interest in the equipment embarrasses the child”), the caregivers did not report these aspects as a negative effect. This may be due to the easier adaptation to changes in the youngest age group, which is rarely affected by appearance-related psychological problems. The dissatisfaction was also caused by the incompatibility of readings between CGM and glucose meter measurements. This discrepancy comes from the delay of sensor readings in relation to the measurement from the glucometer, from the acceptable error of the glucometer, and other factors influencing the imperfection of this method of measuring glycemia. Therefore, it is important to educate about the advantages and disadvantages of CGM. Proper education means avoiding disappointment.

An undeniable benefit, however, remains the significant reduction in the fingerstick glycemia measurements, confirmed by a previous study in our children’s clinic, where the number of fingerstick glycemia measurements was reduced on average by 50% (from 12 to 3 measurements per day at most) [24]. In addition, the current analysis revealed a very high sense of security when using a CGM system compared to the era without CGM.

Slightly more than 50% of the respondents reported that a child was more willing to integrate with peers, was calmer, and was more engaged in the process of self-monitoring. These results are confirmed by the literature data, where as many as 46% of the respondents noticed greater involvement of children in disease control after using the 640G pump-integrated CGM system, although the children were older (average age 8 years) [24].

## 5. Conclusions

The use of rtCGM in children with T1D, especially in the youngest, leads to a high quality of life and an increased quality of sleep in their caregivers.

## Figures and Tables

**Table 1:** Group characteristics.

Total Amount of Children (nr)	38
Age (years)	34% aged 2–4 66% aged 5–7
Average T1D duration (years)	2.6 ± 1.6
Duration of CGM use (years)	1.92 ± 1.15
Average HbA1c (%)	6.53 ± 0.63
Children treated with CGM since the beginning of the disease diagnosis (%)	31.5

T1D—type 1 diabetes, CGM—continuous glucose monitoring.

**Table 2:** The average number of points in the Quality of Life Survey (PedsQL 3.0) in each category.

Communication	Anxiety	Treatment	Diabetes-Related Problems
200/300 (67%)	79/300 (26%)	719/1100 (65%)	723/1100 (66%)

**Table 3:** The average number of points in the Quality of Life Survey in individual categories (PedsQL 3.0).

Communication	Worries	Treatment	Problems Associated with Diabetes
75%	30%	70%	65%

**Table 4:** The quality of life (QoL) Scale.

Very Low	Low	Moderate	High	Very High
0–19%	20–39%	40–59%	60–79%	80–100%

**Table 5:** PedsQL 3.0—most frequently reported concerns.

Concerns about Late Complications of Diabetes	Injection Pain (Injections/Vein Puncture)	Anxiety about Hypoglycemia	Fear of Treatment Failure
Almost always, often 76%	Almost always 30%	Almost always, often 60%	Almost always, often 60%

## Data Availability

The authors confirm that the data supporting the findings of this study are available within the article. The detailed database is available on request from the corresponding author.

## Supplementary Materials

Supplementary Materials are available online at https://www.mdpi.com/article/10.3390/s21113683/s1.

## References

[1] Phillip M., Danne T., Shalitin S., Buckingham B., Laffel L., Tamborlane W., Battelino T., Consensus Forum Participants (2012). Use of continuous glucose monitoring in children and adolescents *. Pediatr. Diabetes.

[2] Kubiak T., Mann C.G., Barnard K.C., Heinemann L. (2016). Psychosocial Aspects of Continuous Glucose Monitoring. J. Diabetes Sci. Technol..

[3] Valenzuela J.M., Patino A.M., McCullough J., Ring C., Sanchez J., Eidson M., Nemery R., Delamater A.M. (2006). Insulin Pump Therapy and Health-Related Quality of Life in Children and Adolescents with Type 1 Diabetes. J. Pediatr. Psychol..

[4] Gawłowicz K. (2012). Assessment of the quality of life of children and young people with type 1 diabetes. Hygeia Public Health.

[5] Hommel E., Olsen B., Battelino T., Conget I., Schütz-Fuhrmann I., Hoogma R., Schierloh U., Sulli N., Gough H., Castañeda J. (2014). Impact of continuous glucose monitoring on quality of life, treatment satisfaction, and use of medical care resources: Analyses from the SWITCH study. Acta Diabetol..

[6] Naranjo D., Hood K. (2013). Psychological challenges for children living with diabetes. Diabetes Voice.

[7] Lal R.A., Maahs D.M. (2017). Clinical Use of Continuous Glucose Monitoring in Pediatrics. Diabetes Technol. Ther..

[8] Deiss D., Bolinder J., Riveline J.-P., Battelino T., Bosi E., Tubiana-Rufi N., Kerr D., Phillip M. (2006). Improved Glycemic Control in Poorly Controlled Patients with Type 1 Diabetes Using Real-Time Continuous Glucose Monitoring. Diabetes Care.

[9] Hawkes C.P., McDarby V., Cody D. (2014). Fear of hypoglycemia in parents of children with type 1 diabetes. J. Paediatr. Child Health.

[10] Cyganek K., Małecki M.T. (2010). Continuous glucose monitoring in patients with diabetes—A review of available systems. Diabetol. Prakt..

[11] New J.P., Ajjan R., Pfeiffer A.F.H., Freckmann G. (2015). Continuous glucose monitoring in people with diabetes: The randomized controlled Glucose Level Awareness in Diabetes Study (GLADIS). Diabet. Med..

[12] Deiss D., Hartmann R., Hoeffe J., Kordonouri O. (2004). Assessment of glycemic control by continuous glucose monitoring system in 50 children with type 1 diabetes starting on insulin pump therapy. Pediatr. Diabetes.

[13] Battelino T., Conget I., Olsen B., Schütz-Fuhrmann I., Hommel E., Hoogma R., Schierloh U., Sulli N., Bolinder J., The SWITCH Study Group (2012). The use and efficacy of continuous glucose monitoring in type 1 diabetes treated with insulin pump therapy: A randomised controlled trial. Diabetology.

[14] Streisand R., Monaghan M. (2014). Young Children with Type 1 Diabetes: Challenges, Research, and Future Directions. Curr. Diabetes Rep..

[15] Rodbard D. (2017). Continuous Glucose Monitoring: A Review of Recent Studies Demonstrating Improved Glycemic Outcomes. Diabetes Technol. Ther..

[16] Emmanouilidou E., Galli-Tsinopoulou A., Karavatos A., Nousia-Arvanitakis S. (2008). Quality of life of children and ado-lescents with diabetes of Northern Greek origin. Hippokratia.

[17] Diabetes Research in Children Network (Direcnet) Study Group (2006). The Diabetes Research in Children Network (DirecNet) Study Group* Psychological aspects of continuous glucose monitoring in pediatric type 1 diabetes. Pediatr. Diabetes.

[18] Varni J.W., Burwinkle T.M., Jacobs J.R., Gottschalk M., Kaufman F., Jones K.L. (2003). The PedsQLTM in Type 1 and Type 2 diabetes: Reliability and validity of the Pediatric Quality of Life Inventory TM Generic Core Scales and Type 1 Di-abetes Module. Diabetes Care.

[19] Nieuwesteeg A., Pouwer F., Van Der Kamp R., Van Bakel H., Aanstoot H.-J., Hartman E. (2012). Quality of life of children with type 1 diabetes: A systematic review. Curr. Diabetes Rev..

[20] Van Name M.A., Hilliard M.E., Boyle C.T., Miller K.M., DeSalvo D.J., Anderson B.J., Laffel L.M., Woerner S.E., DiMeglio L.A., Tamborlane W.V. (2018). Nighttime is the worst time: Parental fear of hypoglycemia in young children witg type 1 diabetes. Pesiatr. Diabetes.

[21] Burckhardt M.-A., Fried L., Bebbington K., Hancock M., Nicholas J.A., Roberts A., Abraham M.B., Davis E.A., Jones T.W. (2018). Use of remote monitoring with continuous glucose monitoring in young children with Type 1 diabetes: The parents’ perspective. Diabet. Med..

[22] Sinisterra M., Hamburger S., Tully C., Hamburger E., Jaser S., Streisand R. (2020). Young Children with Type 1 Diabetes: Sleep, Health-Related Quality of Life, and Continuous Glucose Monitor Use. Diabetes Technol. Ther..

[23] Driscoll K.A., Raymond J., Naranjo D., Patton S.R. (2016). Fear of Hypoglycemia in Children and Adolescents and Their Parents with Type 1 Diabetes. Curr. Diabetes Rep..

[24] Tabor A., Gaweł W.B., Goik O., Deja G., Jarosz-Chobot P. (2017). Evaluation of the quality of life and satisfaction with the therapy in patients with type 1 diabetes—Is Medtronic MiniMed 640G system able to improve it? Preliminary insights. Clin. Diabetol..

