# Supplementary material for: The Quality of Life and Satisfaction with Continuous Glucose Monitoring Therapy in Children under 7 Years of Age with T1D Using the rtCGM System Integrated with Insulin Pump—A Caregivers Point of View
Source: Sensors (Basel). 2021 May 25;21(11):3683. doi: 10.3390/s21113683 (PMC8198889; doi:10.3390/s21113683)
Supplement: Supplementary file 1 [file sensors-21-03683-s001.zip › sensors-1203578-supplementary.pdf]

Dear Sir/Madame

We kindly ask you to complete a quality-of-life questionnaire regarding your child's continuous glucose monitoring system (CGM) use. The obtained results will broaden the knowledge about the effectiveness of the CGM system in children.

1. **How long has the child been using the CGM system?**

2. **How long after the diagnosis of diabetes mellitus type 1 (DM1) did the child start using the CGM system?**

3. **The last HbA1c:**

date: ………………………………………………………………………………………….

result: …………………………………………………………………………………………

4. **Did your child experience an episode of severe hypoglycaemia (loss of consciousness, needing glucagon) while using the CGM system?**

A: Yes

If yes, how many times? ....

B: No.

5. **How CGM system affected the child's functioning in the kindergarten / school?**

<u>Had a positive effect:</u>

A. The child is calmer

B. The child is more willing to play with peers

C. The child is more willing to engage in sports activities

D. Willingly undertakes additional activities

E. The child is more engaged in diabetes self-control

F. Others: ....

<u>Had a negative effect:</u>

A. The child is ashamed of having another piece of equipment on the body, feeling stigmatized by the disease

B. The child is afraid to start a sport activity because of fear of damaging equipment.

C. The child withdraws from relationships with his peers

D. Peers are afraid to play with the child because of the presence of various "devices" on his body.

E. CGM do not allow the caregivers to focus on anything else.

F. The constant alarms keep your child from paying attention to them anymore.

G. Other ....

<u>Neutral</u>

6. How does the kindergarten / school staff react to the child's use of CGM?

<u>Positively</u>

A. The kindergarten / school staff is more willing to look after the child

B. The staff is calmer and more confident to work with the child

C. Other………………………………………………………………………………………..

<u>Negatively</u>

A. Kindergarten / school staff is concerned about the use of new technical equipment by the child.

B. The staff is concerned about damaging the equipment

C. The staff feels plagued by the disease, is under pressure from CGM

D. Other ……………………………………………………………………………………..

Neutral

7. **Does the child stay under the care of other people more often than before since CGM started?**

A. Yes

B. No.

8. **Has your child started extra sports activities since starting CGM?**

A. Yes

B. No.

9. **Does CGM affect the caregiver's sleep quality?**

Has a positive effect

A. I sleep better, I am calmer

B. Others ……………………………………………………………………………………….

Has a negative effect

A. alarms wake me up at night

B. Others ……………………………………………………………………………………….

Neutral

10. **Does CGM affect the child's sleep quality?**

Has a positive effect

A. The child sleeps more peacefully

B. Other …………………………………………… ....

<u>Has a negative effect</u>

A. Alarms wake your child up from sleep

B. Others……………………………………………………………………………………

<u>Neutral</u>

11. **Has the frequency of fingerstick blood glucose measurements CHANGED while using the CGM?**

A: Yes

number of measurements before the CGM system ....

during the use of CGM ....

B: No.

12. **How do you assess the child's safety regarding hypo and hyperglycaemia in the following situations - please rate it on the following scales:**

A: <u>When using CGM</u>

High level of danger 1 2 3 4 5 complete safety

B: <u>Without CGM system:</u>

High level of danger 1 2 3 4 5 complete safety

13. **Has the CGM system changed any of the following spheres of family life? Select all that you observe and list some examples of changes:**

A: changes in family eating habits: ....

....

....

....

....

B: changes in the style of spending free time (e.g. holidays, trips)

....

....

....

....

....

....

C: changes in parents' working life

....

....

....

....

....

....

D: no changes in the above areas

4. Are you satisfied with your child's treatment with CGM use? Does CGM meet your expectations?

A: Yes because

....

....

....

....

....

....

B: No, because

....

....

....

....

....

....

C: It's hard to say because

....

....

....

....

....

....

Thank you for completing the survey!
